# Endovascular treatment after the fenestrated frozen elephant trunk technique

**DOI:** 10.1002/ccr3.6595

**Published:** 2022-11-15

**Authors:** Satoshi Okugi, Masaaki Koide, Yoshifumi Kunii, Minori Tateishi, Risa Shimbori, Hiroki Moriuchi, Masataka Hayashi

**Affiliations:** ^1^ Department of Cardiovascular Surgery Seirei Hamamatsu General Hospital Shizuoka Japan; ^2^ Department of Neurosurgery Seirei Hamamatsu General Hospital Shizuoka Japan

**Keywords:** dissecting aneurysm, endoleak, endovascular procedures, thoracic aorta, vertebral artery

## Abstract

Recently, several centers have performed total arch replacement using the fenestrated frozen elephant trunk technique for acute Stanford type‐A aortic dissection. However, the long‐term results and need for additional treatment following this procedure are unclear. We report a case of a 54‐year‐old man who underwent endovascular therapy for endoleaks after total arch replacement using the fenestrated frozen elephant trunk technique for acute type‐A aortic dissection with an isolated left vertebral artery. After the surgery, the endoleak was resolved, and the patient was asymptomatic with no neurological deficits. This strategy might be effective in similar cases.

## Abstract 2

When endoleak occur after TAR using the fenestrated frozen elephant trunk technique in patients with rare anatomical subtypes, collaboration with other experts facilitates additional treatment.

## 1 INTRODUCTION

As a surgical treatment for acute Stanford type‐A aortic dissection, total arch replacement (TAR) using the frozen elephant trunk (FET) technique is being performed in several institutions.^1^ Recently, favorable surgical outcomes after fenestrated FET for acute type‐A aortic dissection have been reported.^2^ However, reports describing the need for additional treatment after surgical treatment of acute aortic dissection with fenestrated FET are few.^2^ We report the successful use of endovascular surgery to treat an endoleak following fenestrated FET for acute type‐A aortic dissection.

## 2 CASE

**FIGURE 1:**
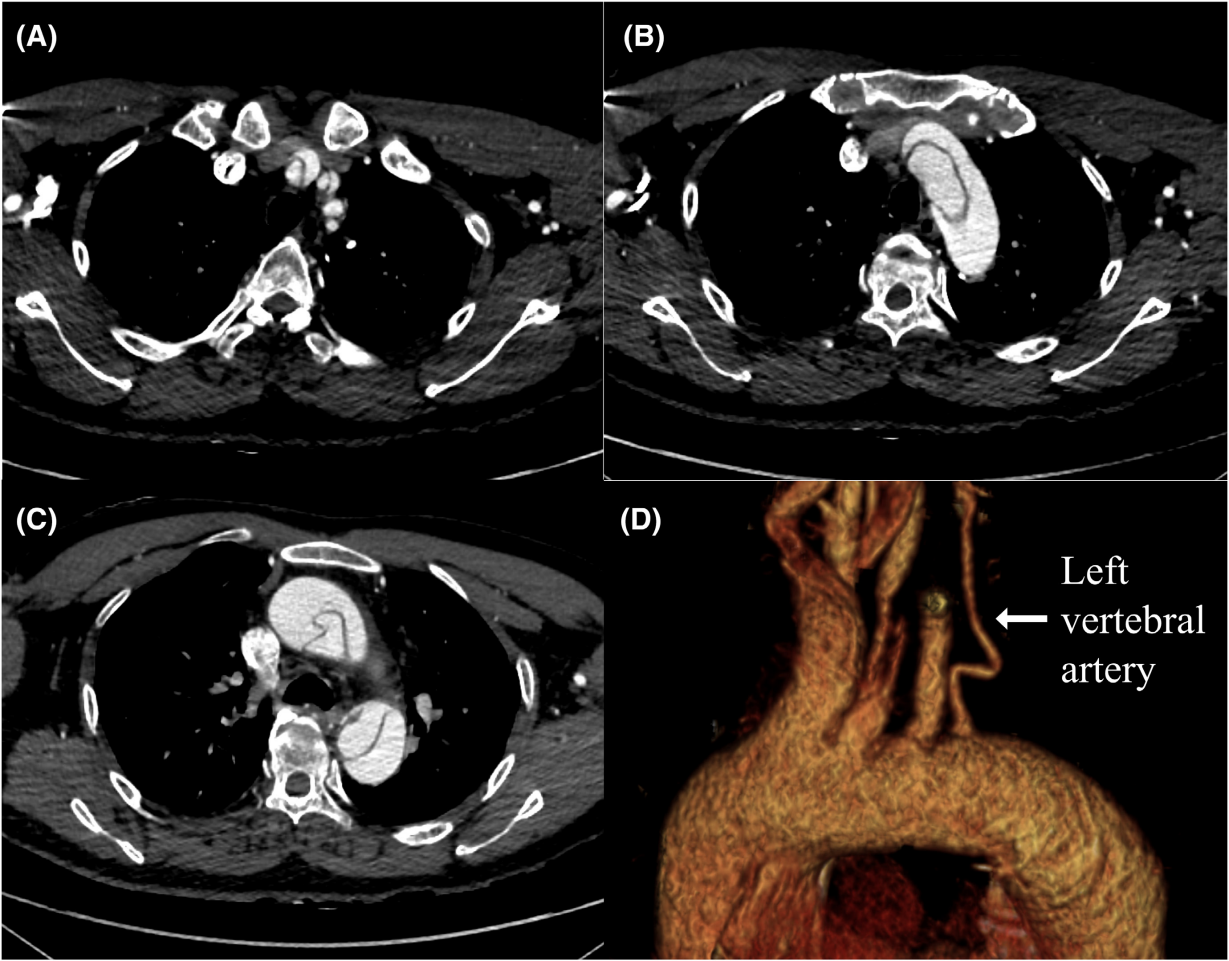
(A,B,C) Preoperative computed tomography showing a Stanford type‐A aortic dissection with an entry in the aortic arch. (d) Preoperative computed tomography showing an isolated left vertebral artery distal to the left subclavian artery

A 54‐year‐old man was transferred to our hospital because of chest pain and left hemiparesis. His medical history included untreated hypertension. He had no family history of aortic disease. Computed tomography (CT) revealed a Stanford type‐A aortic dissection with an entry in the aortic arch and an isolated left vertebral artery distal to the left subclavian artery (LSCA) (Figure 1).

**FIGURE 2:**
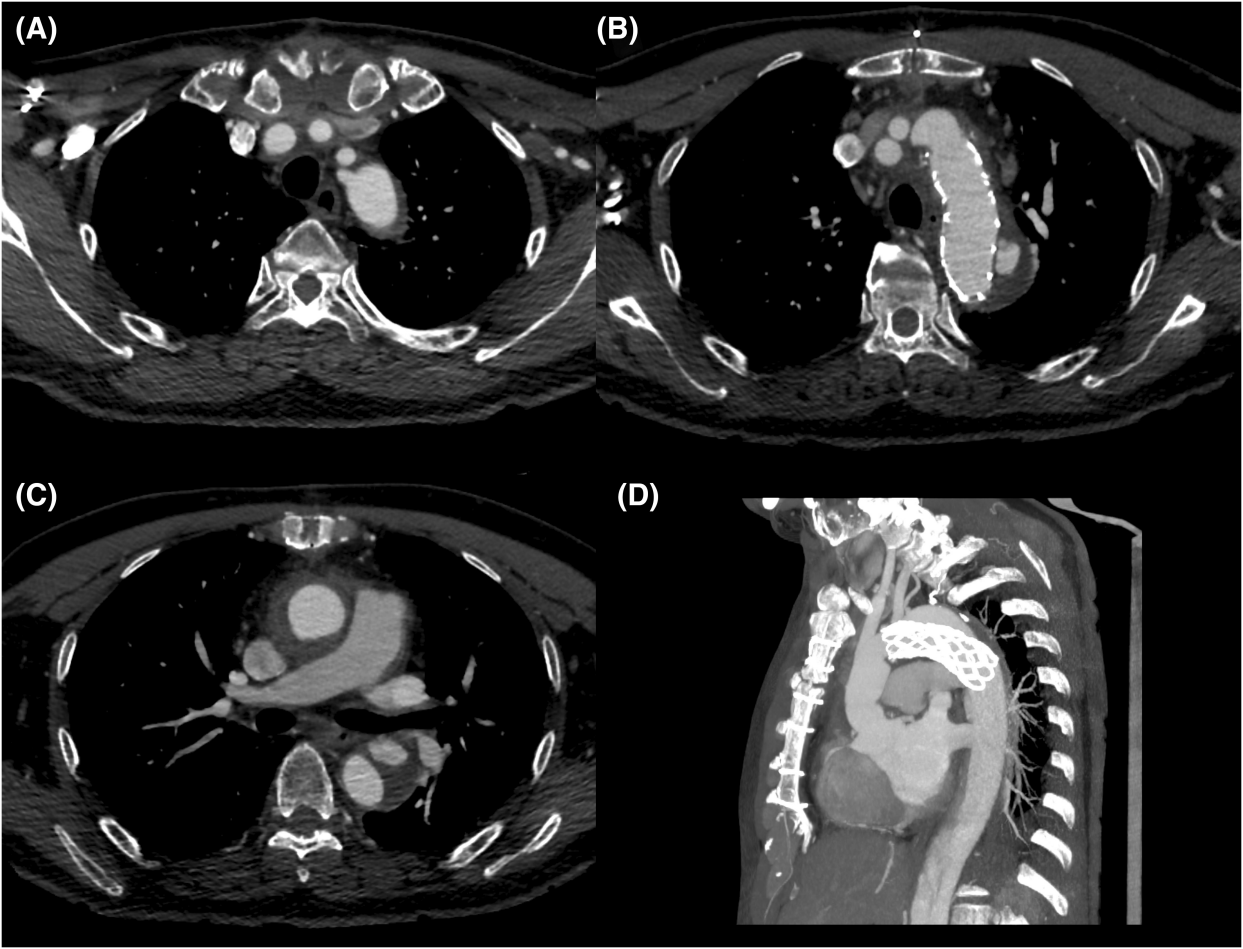
(A,B,C,D) Postoperative computed tomography showing that the antegrade blood flow into the left subclavian artery from the fenestration in the frozen elephant trunk was preserved. Type‐2 endoleak from left vertebral artery to the false lumen in the aortic arch is shown, and endoleak from the fenestration is suspected

**FIGURE 3:**
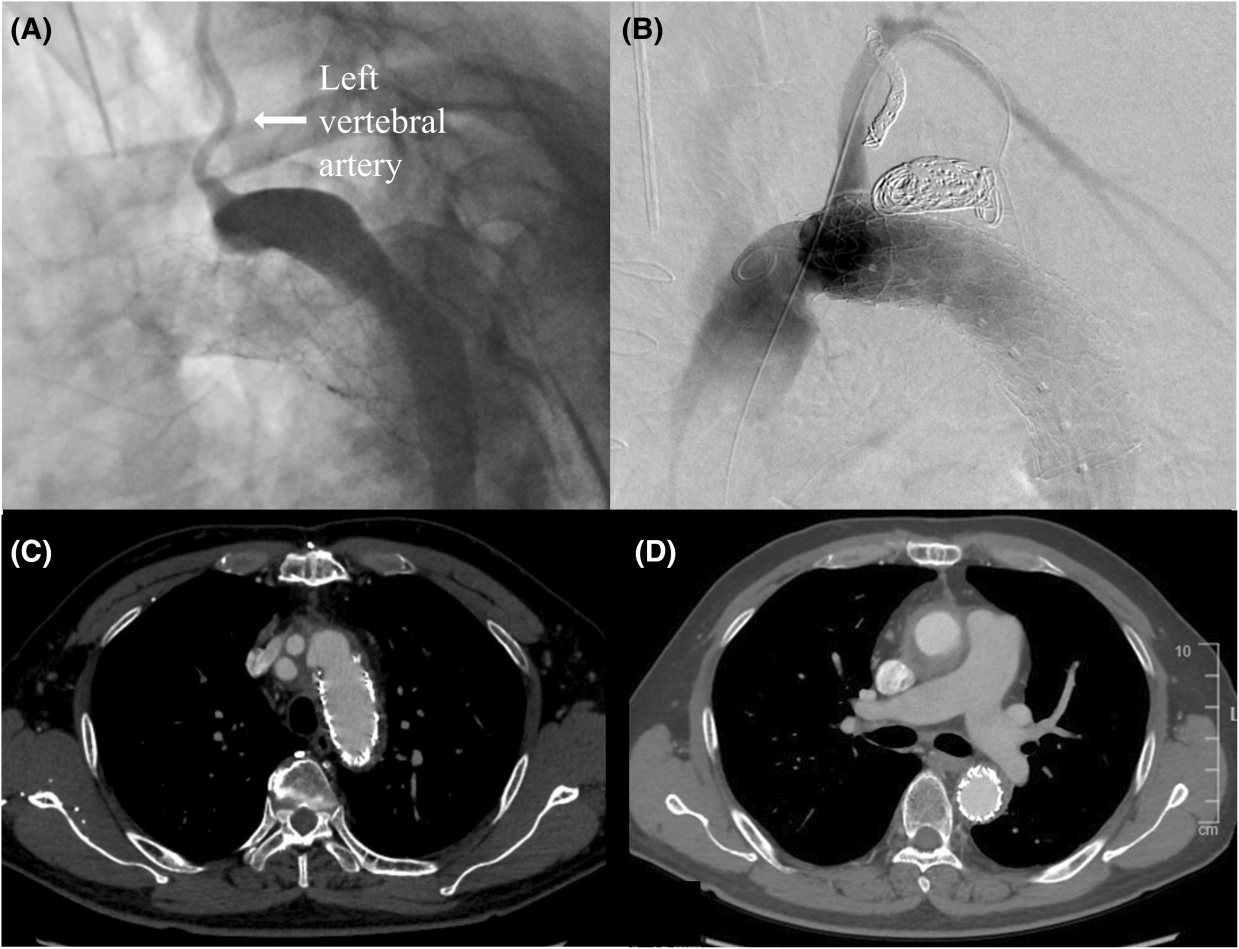
(A) Intraoperative angiography showing a patent left vertebral artery. (B) Post‐treatment angiography reveals that the antegrade blood flow into the left subclavian artery from the fenestration in the frozen elephant trunk is preserved and no endoleaks. (C,D) A 1 year post‐treatment computed tomography shows the false lumen had shrunken

An emergency surgery was performed through median sternotomy. Cardiopulmonary bypass was performed using the right femoral artery (FA) and bicaval cannulation and aortic cross‐clamping. The ascending aorta was transected, and unbranched portion of a 24 mm four‐branched J‐graft (Japan Lifeline, Tokyo, Japan) was anastomosed to the proximal side of the ascending aorta with 4–0 polyvinylidene difluoride sutures using a felt strip. Antegrade‐selective cerebral perfusion was started. An intimal tear was identified on the distal lesser curvature side of the aortic arch. A 25‐ × 90 mm Frozenix (Japan Lifeline)^3^ was inserted into the true lumen and deployed. Two 5 mm holes were created using electrocautery in the stent portion of the Frozenix in the area where the LSCA and left vertebral artery met. A four‐branched J‐graft (Japan Lifeline) was anastomosed to the transected aorta with 4–0 polyvinylidene difluoride sutures using a felt strip. The aortic cross‐clamping and total circulatory arrest times were 143 and 38 min, respectively. The postoperative course was uneventful. Postoperative CT revealed preserved antegrade blood flow to the LSCA from fenestration in the FET; however, a type‐II endoleak from the detached left vertebral artery to the false lumen in the aortic arch was observed. Further, an endoleak from the fenestration was suspected (Figure 2). Thus, additional treatment was performed. The surgical plan included coil embolization of the left vertebral artery and thoracic endovascular aortic repair (TEVAR) to terminate endoleaks from the fenestration site. We consulted a neurosurgeon to confirm that the arterial ring of Willis was well developed using contrast CT angiography before the surgery. Three months after the initial aortic dissection, endovascular surgery was performed with the patient under general anesthesia. The left FA was exposed. A 9‐French (Fr) short sheath was placed in the left FA and a 6‐Fr long sheath in the right FA. A hybrid operating room (Artis Zeego; SIEMENS, Munich, Germany) was used for angiography. A Goodtec angiographic catheter and Radifocus guidewire (Terumo, Tokyo, Japan) were inserted from the right FA to the false lumen in the aortic arch through re‐entry from the true lumen of the abdominal aorta. A microcatheter was inserted into the left vertebral artery (Figure 3A). Galaxy G3 microcoils (Johnson & Johnson, New Brunswick, NJ, USA), Target 360 microcoils (Stryker, Kalamazoo, MI, USA), and Axium PRIME microcoils (Medtronic, Dublin, IE) were used for embolization of the left vertebral artery by a neurosurgeon. A 20‐Fr DrySeal Flex Introducer Sheath (WL Gore & Associates Inc., Delaware, USA) was placed in the left FA. The Conformable GORE TAG Thoracic Endoprosthesis (TGU312610J; WL Gore & Associates Inc.) was inserted from the left FA and deployed after ensuring it was in the correct position to cover the fenestration for left vertebral artery using contrast imaging. Interlock microcoils (Boston Scientific, Marlborough, MA, USA) were used for embolization of the false lumen in the aortic arch. A final imaging procedure was conducted to ensure that there were no endoleaks and that LSCA blood flow was preserved so that the surgery could be completed (Figure 3B). The patient's postoperative course was uneventful, and he had no neurological deficits. Postoperative CT revealed occlusion of the left vertebral artery, no endoleak, and patency of the fenestration for the LSCA in the FET. The patient was discharged 8 days postoperatively and has been asymptomatic for 18 months postoperatively. A 1 year postoperative CT revealed the false lumen had shrunken with fenestration patency for the LSCA in the FET (Figure 3C,D).

## 3 DISCUSSION

The fenestrated FET technique can simplify neck vessel reconstruction in TAR and decrease the aortic cross‐clamping and circulatory arrest times.^2^ However, complications include endoleaks from fenestration.^2^ TAR using the FET technique for acute type‐A aortic dissection has favorable early and late postoperative outcomes.^4^, ^5^ Endovascular interventions after FET have often been reported.^6^ Recently, early and mid‐term results of the fenestrated FET technique for treating acute type‐A aortic dissection were reported.^2^ In our case, the left vertebral artery originated independently distal to the LSCA, which is a rare anatomical finding. TAR using the fenestrated FET technique was performed; however, an endoleak from the left vertebral artery, which was detached from the true lumen, was observed. Therefore, additional treatment was performed to prevent aneurysmal dilatation in the aorta. The support of expert neurosurgeons skilled in vertebral artery coil embolization allowed for a safe and smooth endovascular procedure. The patient underwent coil embolization and TEVAR 3 months after the onset of aortic dissection, with no neurological deficit or stent graft‐induced new entry. With appropriate case selection, fenestrated FET has the potential to be an effective treatment for acute aortic dissection, although the complications of endoleak and occlusion of the fenestration have not been solved completely. A long‐term follow‐up assessment of the fenestration‐site aortic remodeling of the false lumen and neurological findings is required to evaluate the future applicability of these findings.

## 4 CONCLUSION

This report suggests that when endoleak occur in patients with rare anatomical subtypes after TAR using the fenestrated FET technique, collaboration with an expert neurosurgeon in endovascular treatment facilitates appropriate additional treatment.

## AUTHOR CONTRIBUTIONS

Satoshi Okugi and Masaaki Koide involved in conceptualization, data curation, writing the original draft, review, and editing. Yoshifumi Kunii, Minori Tateishi, Risa Shimbori, and Hiroki Moriuchi involved in data curation, review and editing. Masataka Hayashi involved in conceptualization, review and editing.

## CONFLICT OF INTEREST

The authors report no conflict of interest.

## ETHICAL APPROVAL

None.

## CONSENT

Written informed consent was obtained from the patient to publish this report in accordance with the journal's patient consent policy.

## Data Availability

The data that support the findings of this study are available from the corresponding author upon reasonable request.
